# Artificial Intelligence in Primary Malignant Bone Tumor Imaging: A Narrative Review

**DOI:** 10.3390/diagnostics15131714

**Published:** 2025-07-04

**Authors:** Platon S. Papageorgiou, Rafail Christodoulou, Panagiotis Korfiatis, Dimitra P. Papagelopoulos, Olympia Papakonstantinou, Nancy Pham, Amanda Woodward, Panayiotis J. Papagelopoulos

**Affiliations:** 1 First Department of Orthopaedics, University General Hospital Attikon, Medical School, National and Kapodistrian University of Athens, 12462 Athens, Greecepjportho@med.uoa.gr (P.J.P.); 2 Department of Radiology, School of Medicine, Stanford University, Stanford, CA 94305, USA; 3 Department of Radiology, Mayo Clinic, Rochester, MN 55905, USA; korfiatis.panagiotis@mayo.edu; 4 Second Department of Radiology, University General Hospital Attikon, Medical School, National and Kapodistrian University of Athens, 12462 Athens, Greece; sogofianol@gmail.com

**Keywords:** primary bone tumors, machine learning, imaging, radiomics, orthopedic oncology

## Abstract

Artificial Intelligence (AI) has emerged as a transformative force in orthopedic oncology, offering significant advances in the diagnosis, classification, and prediction of treatment response for primary malignant bone tumors (PBT). Through machine learning and deep learning techniques, AI leverages computational algorithms and large datasets to enhance medical imaging interpretation and support clinical decision-making. The integration of radiomics with AI enables the extraction of quantitative features from medical images, allowing for precise tumor characterization and the development of personalized therapeutic strategies. Notably, convolutional neural networks have demonstrated exceptional capabilities in pattern recognition, significantly improving tumor detection, segmentation, and differentiation. This narrative review synthesizes the evolving applications of AI in PBTs, focusing on early tumor detection, imaging analysis, therapy response prediction, and histological classification. AI-driven radiomics and predictive models have yielded promising results in assessing chemotherapy efficacy, optimizing preoperative imaging, and predicting treatment outcomes, thereby advancing the field of precision medicine. Innovative segmentation techniques and multimodal imaging models have further enhanced healthcare efficiency by reducing physician workload and improving diagnostic accuracy. Despite these advancements, challenges remain. The rarity of PBTs limits the availability of robust, high-quality datasets for model development and validation, while the lack of standardized imaging protocols complicates reproducibility. Ethical considerations, including data privacy and the interpretability of complex AI algorithms, also warrant careful attention. Future research should prioritize multicenter collaborations, external validation of AI models, and the integration of explainable AI systems into clinical practice. Addressing these challenges will unlock AI’s full potential to revolutionize PBT management, ultimately improving patient outcomes and advancing personalized care.

## 1. Introduction

**Figure 1:**
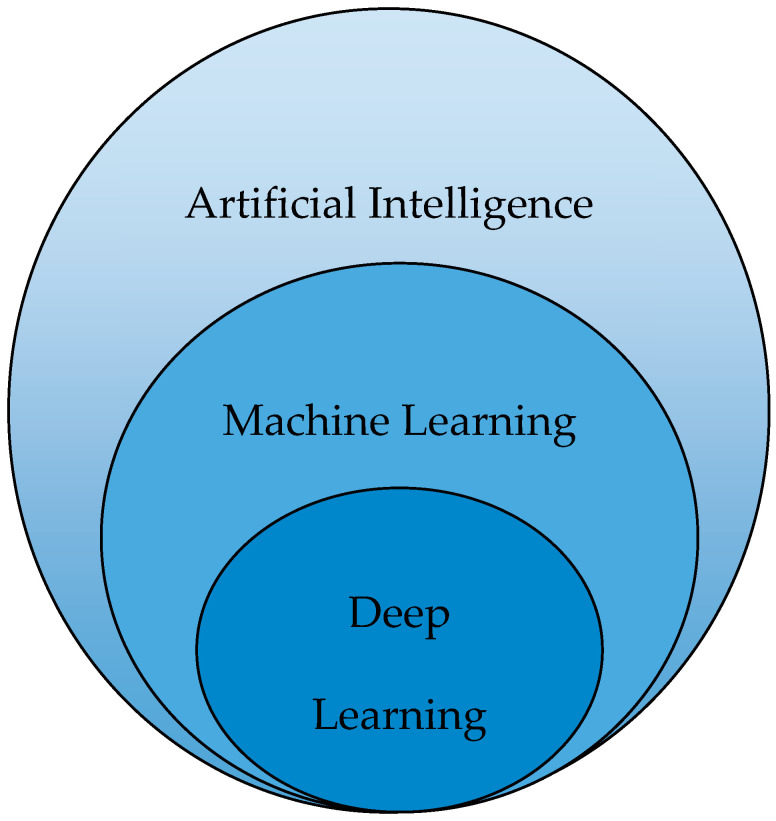
The correlations among artificial intelligence, machine learning, and deep learning.

The term AI refers to a field of computer science that enables computers to function like human cognitive processes. It was first introduced by McCarthy in the 1950s, with the first FDA-approved AI algorithm occurring in 2017. A subset of AI, machine learning (ML), employs computational algorithms that enhance task performance through experience. Over the years, advancements in computational algorithms, combined with the rise of big data, have led to the development of deep learning, a specialized subset of machine learning. Deep learning (DL) uses artificial neural networks that mimic the architecture of biological nervous systems. Among these, convolutional neural networks (CNN) have gained popularity in radiology due to their outstanding capabilities in imaging pattern recognition [1], (Figure 1). Radiomics involves extracting mathematical features from medical images [2]. The integration of radiomics with machine learning facilitates the identification of complex patterns for diagnosis, prognosis prediction, classification, and treatment response assessment in orthopedic oncology [3]. Despite the low incidence of PBT, accurate diagnosis and classification are vital due to their variable biological behaviors and treatment needs [4]. Radiomics–ML has shown promising results in analyzing lesion characteristics, standardizing image comparisons, and enhancing diagnostic accuracy. Research has also shown the potential of AI tools in differentiating specific tumor types, such as enchondroma and chondrosarcoma, using MRI-based radiomic features [5].

Indeed, MRI is the most sensitive modality for evaluating primary bone tumors, as it allows for the assessment of bone marrow involvement, soft tissue invasion, and lesion fluid content. Radiologists encounter numerous diagnostic dilemmas; for instance, distinguishing primary bone tumors from bone infections can be quite challenging due to overlapping features [4].

Radiomics texture feature extraction entails the quantitative analysis of medical images to capture the underlying tissue heterogeneity within a defined region of interest (ROI), such as a tumor or lesion [6]. This process typically commences with the acquisition of high-quality images, such as MRI or CT scans, followed by the segmentation of the ROI, either manually by experts or through automated methods. Once the ROI is established, the image data is preprocessed to ensure consistency and reliability in feature extraction. This may include steps like intensity normalization, resampling to a standard voxel size, and discretizing grayscale values into fixed bins to minimize noise and enhance pattern detection [7].

Texture features are then extracted using statistical matrices that describe the spatial relationships between pixel or voxel intensities. One of the most commonly used is the Gray-Level Co-Occurrence Matrix (GLCM), which quantifies how often pairs of pixel intensities occur at a given distance and orientation. Other widely used matrices include the Gray-Level Run Length Matrix (GLRLM), which captures the length of consecutive pixels with the same intensity, and the Gray-Level Size Zone Matrix (GLSZM), which measures the size of homogeneous intensity zones within the ROI [8].

From these matrices, a variety of quantitative features are derived. These include contrast, which measures local intensity variation; entropy, which reflects the complexity or randomness of the texture; homogeneity, which indicates how uniform the intensity distribution is; energy, a measure of image uniformity; and correlation, which assesses the linear dependency of gray levels across pixels.

This narrative review discusses in detail the applications of AI in PBT, including tumor detection, imaging processing, therapy response prediction, and tumor classification. While ML and DL techniques continue to evolve, their application to PBTs is still in its early stages. The development of advanced DL models capable of simultaneous detection, segmentation, and classification represents a significant step forward in the field [9]. By the integration of artificial intelligence in clinical practice, we can potentially improve clinical decision-making, personalize treatment strategies, and finally improve patient outcomes. Through this review, we also aim to highlight the current advancements, practical applications, and future directions of artificial intelligence in managing PBT [2,5]. A key distinction of this review is its emphasis on synthesizing both qualitative and quantitative aspects of AI applications across the entire spectrum of PBT imaging, including the latest advancements in simultaneous detection, segmentation, and classification using advanced DL models. Unlike prior reviews, which often concentrate on a single imaging modality or focus narrowly on diagnostic accuracy, this review systematically summarizes the strengths and limitations of current AI approaches, discusses the methodological quality and heterogeneity of included studies, and explicitly addresses the clinical and technical barriers to implementation.

## 2. Materials and Methods

An extensive search of the Medline/PubMed, Embase, and Scopus libraries in the English literature was conducted in December 2024. For the search we used the keywords “artificial intelligence”, “Neural Networks, Computer”, “Image Processing, Computer-Assisted”, “Deep Learning”, “Machine Learning”, “Artificial Intelligence”, “Artificial Neural Network”, “Convolutional Neural Network”, “Deep Learning”, “Machine Learning”, “image processing”, “automated detection”, “Diagnostic Imaging”, “diagnostic imaging”, “imaging”, “Osteosarcoma”, “Chondrosarcoma”, “Histiocytoma, Malignant Fibrous”, “Fibrosarcoma”, “Osteosarcoma”, “Osteosarcomas”, “Osteogenic Sarcoma”, “Osteogenic Sarcomas”, “Osteosarcoma Tumor”, “Osteosarcoma Tumors”, “Ewing Sarcoma”, “Ewing Tumor”, “Chondrosarcoma”, “Chondrosarcomas”, “Malignant Fibrous Histiocytomas”, “Malignant Fibrous Histiocytoma”, “Malignant Fibrohistiocytic Tumor”, “Malignant Fibrohistiocytic Tumors”, “Fibrosarcoma”, “Fibrosarcomas”, “Bone Lymphoma”, “primary”, “bone cancer”, “bone neoplasm”, “bone cancers”, “bone neoplasms”, “bone tumor”, “bone tumors”, “bone metastasis”. The studies were eligible as long as they included any type of primary bone tumor and any tool of AI; all types of studies, except conference abstracts, from 2017 to 2024 were deemed eligible. Also, the studies had to be focused only on the human population.

**Figure 2:**
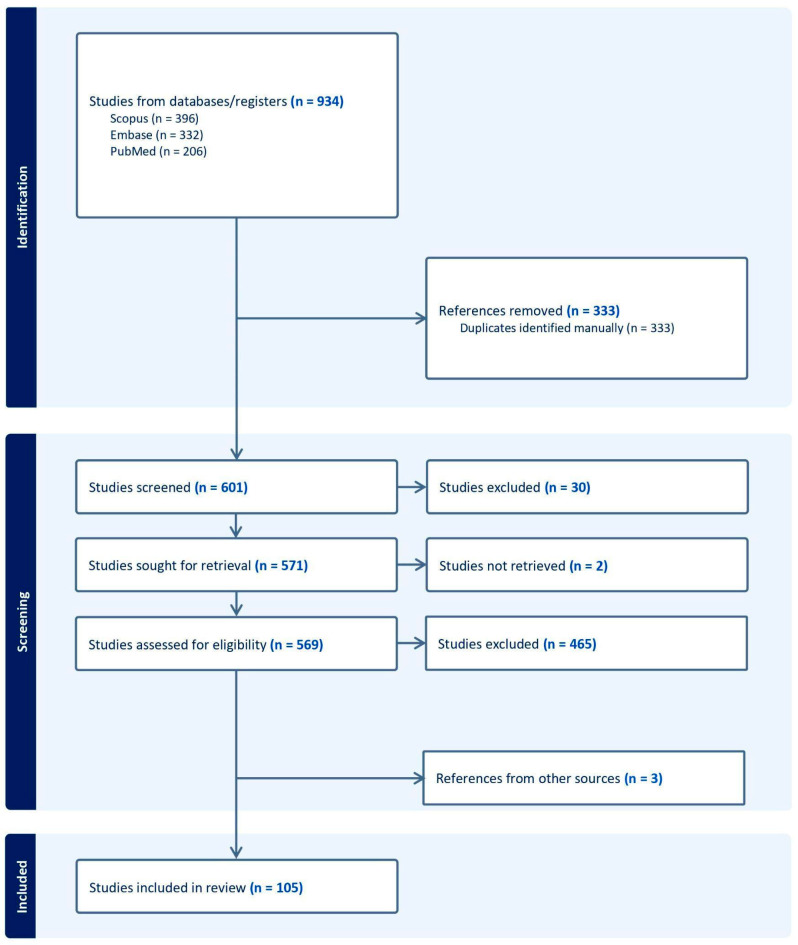
The workflow of search and selection. The search was conducted in December of 2024.

The abstract screening was performed by the primary authors, while full-text screening was conducted collaboratively by all authors. Final inclusion decisions were made by consensus among all authors to ensure the relevance and high quality of the selected data. A total of 601 records were identified, from which 102 fulfilled our inclusion criteria. The workflow process is demonstrated in Figure 2.

## 3. Results

**Figure 3:**
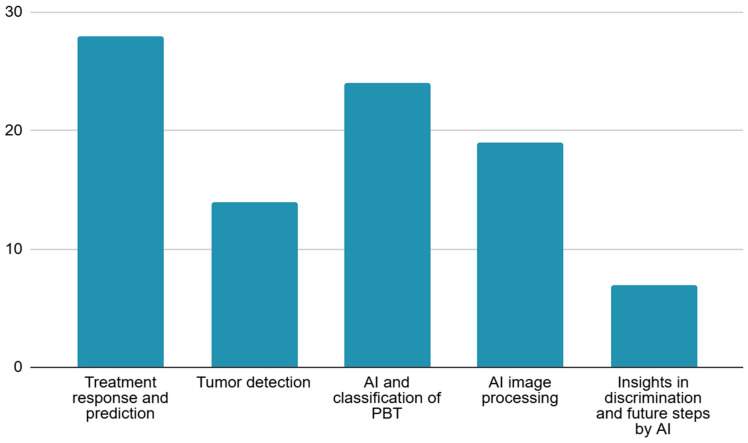
Clustering the studies according to the main area of focus.

A total of 105 articles published from 2017 to 2024 were included in this narrative review after more than 600 high-quality articles were screened. These articles were categorized into five main domains, as demonstrated in Figure 3. The majority of studies focused on osteosarcoma, followed by Ewing sarcoma and chondrosarcoma. Deep learning models, particularly convolutional neural networks (CNNs), were the most commonly applied AI tools, achieving high diagnostic accuracies, often surpassing radiologists in detection and classification tasks. In addition, Radiomics-based machine learning models demonstrated strong predictive power in evaluating chemotherapy response, with area under the curve (AUC) values frequently exceeding 0.85. Furthermore, segmentation models using advanced architectures such as U-net, transformers, and hybrid CNN–transformer systems reported Dice similarity coefficients above 0.90, highlighting the efficacy of AI in enhancing diagnostic workflows and reducing physician workload.

## 4. Discussion

### 4.1. Treatment Response and Prediction

Treatment response and prediction in PBT enhances precision medicine, improves patient outcomes, and reduces the burden of ineffective therapies. The selected studies can be found in Table 1. Radiomics, a method that extracts quantitative features from medical images, has been very promising in chemotherapy response predictions. Studies have compared 2D and 3D MRI radiomics; 2D extracts features from a single or a few selected slices of a volumetric scan (e.g., the largest cross-sectional slice of a tumor), and 3D extracts features from the entire tumor volume (across all slices), capturing spatial heterogeneity in three dimensions [10]. In the case of skeletal Ewing sarcoma, the superior reproducibility of 3D features in predicting responses to neoadjuvant chemotherapy has been demonstrated [11]. Additionally, delta-radiomics models, which evaluate temporal changes in imaging features, have allowed for preoperative assessments of chemotherapy response in high-grade osteosarcoma, having an area under the curve (AUC) of 0.871 in the training cohort and 0.843 in the validation cohort [12].

To standardize these predictions, scoring systems have been developed. A multicenter study introduced a revised scoring system with the assistance of four ML models, logistic regression [LR], decision tree [DT], support vector machine [SVM], and neural network [NN], that could accurately predict neoadjuvant chemotherapy responses in primary high-grade bone sarcomas, achieving an AUC of 0.893 [13]. Furthermore, a deep learning model coupled with an MRI-based radiomics nomogram automated the prediction process of neoadjuvant chemotherapy (NAC) in osteosarcoma patients, providing reliable and efficient evaluations achieving an AUC of 0.793 (95% CI, 0.610–0.975), and the decision curve analysis (DCA) suggested the clinical utility of this nomogram [14].

Preoperative imaging techniques have also benefited from AI integration. A CT-based deep learning radiomics model (DLRM) demonstrated its ability to predict the histological grade and prognosis of chondrosarcoma in comparison to radiomics signature (RS) and deep learning signature (DLS), scoring an AUC of 0.879 with 95% CI, 0.802–0.956 [15]. Similarly, Teo, K. et al. used a support vector machine (SVM) with radial basis function (RBF) for the classification method, combining histopathology data with multi-modal MRI, and found that conventional MRI chemotherapy response predictions in childhood osteosarcoma, by identifying histopathological tumor necrosis, improved by above 95% when dynamic contrast enhanced DCE-MRI was added into consideration [16].

Interactive deep learning tools have made annotation processes, which are a prerequisite for calculating texture features, and CNNs training more efficient. A deep interactive learning approach, deep interactive learning (DIaL), facilitated the rapid labeling of treatment response data for osteosarcoma, reducing the time required for CNN model training to 7 h [17]. Moreover, a Siamese network (DS-Net) effectively differentiated from necrotic tumor regions, streamlining tumor segmentation tasks using hematoxylin and eosin (H&E)-stained osteosarcoma histology slides and achieving an average accuracy of 95.1% [18].

AI models trained on multimodal imaging data have demonstrated enhanced prediction capabilities. A deep learning model using Fluorodeoxyglucose (18F-FDG) positron emission tomography (PET) images found that a deep learning architecture with the selected radiomics feature provides higher prediction accuracy of chemotherapy response in patients with osteosarcoma [19]. A class-structured convolutional neural network applied to diffusion-weighted imaging (DWI), implementing peak signal-to-noise ratio (PSNR), mean square error (MSE), and edge preserve index (EPI) to evaluate the image quality after processing by the CSDCNN algorithm and provided novel insights into osteosarcoma prognosis, scoring better denoising, accuracy, recall, precision, F1 score, and effect evaluation of neoadjuvant chemotherapy with an apparent diffusion coefficient ADCmean value of the patients after chemotherapy of 1.66 ± 0.17 and an ADCmin value of 1.33 ± 0.15 [20]. Directionally sensitive fractal radiomics, applying least absolute shrinkage and selection operator (LASSO) machine learning, revealed associations with chemoresistance in osteosarcoma, with AUCs reaching 0.95, and the capability of handling irregularly shaped tumor regions, in contrast to most radiomic analytical methods, which are compatible only with rectangular regions of interest (ROIs) [21].

Machine learning-based MRI radiomics nomograms have shown significant promise in evaluating chemotherapy efficacy. A DWI-based radiomics model successfully assessed neoadjuvant chemotherapy responses in osteosarcoma patients, outperforming the standalone clinical or radiomics model, attaining an AUC of 0.848 [22]. Combining multi-parametric MRI data with machine learning further enhanced the evaluation of necrosis post-chemotherapy in patients with osteosarcoma, significantly improving discriminating ability for distinguishing, non-cartilaginous tumor survival from tumor nonviable AUC from 0.93 to 0.97, tumor survival from tumor nonviable AUC from 0.83 to 0.9, and cartilaginous tumor survival from tumor nonviable AUC from 0.61 to 0.81 [23].

Fusion radiomics, DLRM, which merges imaging features from multiple modalities, has also been applied to improve predictions of NAC in patients with osteosarcoma. Advances in MRI techniques like the DLRM developed by Zheng et al., which reviewed axial T2-weighted imaging (T2WI) and contrast-enhanced T1-weighted (T1CE), have refined chemotherapy assessments, with dynamic contrast-enhanced MRI models predicting treatment efficacy in osteosarcoma achieving an accuracy of 93.8% and an AUC of 0.961 [7]. Similarly, Zhang, L. et al. utilized K-nearest neighbor (KNN), SVM, and LR for a model establishment to evaluate the value of machine learning-based DCE-MRI radiomics nomogram, attaining an AUC of 0.86, 0.92, and 0.93, respectively [24]. In another study, Zhang, Y. et al. constructed a radiomic model based on before and after NAC, predicting the histological response to NAC in patients with high-grade osteosarcoma using MRI-based radiomics, correlating with improved survival in localized high-grade osteosarcoma, scoring an AUC of 0.999 and 0.915; higher scores were achieved in post-NAC [25]. Texture analysis of intraosseous and extraosseous lesions further contributed to predicting patient outcomes, implementing T2-weighted images in extraosseous with AUCs of 0.94 and 0.89 and T1-weighted images for intraosseous with AUCs of 0.99 and 0.88 [26]. AI’s predictive application extends even further to pediatric sarcomas. For instance, machine learning algorithms trained on MRI-based radiomics effectively predicted neoadjuvant chemotherapy responses in Ewing sarcoma with an AUC of 0.9 [10]. Additionally, Chen et al. selected thirteen radiomics features based on the LASSO-LR classifier to construct the CE FS T1WI radiomics signature that demonstrated potential for forecasting pathological response to NAC in young patients with osteosarcoma [27]. According to Miedler et al., radiomic features in pediatric Ewing sarcoma seem to have the potential to distinguish between children with good and poor response already before and during NAC [28]. Innovative approaches, including the use of infrared spectroscopy combined with machine learning, add new dimensions to treatment outcome prediction for Ewing sarcoma with an accuracy of 92% [29].

Machine learning applied to radiomics data and FDG-PET imaging has further enhanced the prediction of chemotherapy response in osteosarcoma, achieving an AUC-ROC of 0.98, a sensitivity of 100% [30]. Baseline textural features from FDG-PET imaging, analyzed through principal component analysis (PCA) and machine learning linear SVM, offer valuable insights into treatment outcomes as they contribute to better scores in AUC [31]. Pretherapeutic MRI radiomics has demonstrated predictive capabilities for histologic response in osteosarcoma, with the most predictive model achieving an AUC of 0.97 [32], while convolutional neural networks of tumor center FDG-PET images enhance response prediction before chemotherapy in osteosarcoma patients [33].

Binary convolutional neural networks and machine learning techniques trained on PET data continue to improve prediction accuracy for osteosarcoma [34,35]. Prognostic logistic models utilizing metabolic imaging phenotypes further refine response predictions, integrating tumor biology and imaging features to SUVmax and GLZLM_SZLGE (Gray-Level Zone Length based on intensity-size-zone Matrix_Short-Zone Low Grey-Level Emphasis) as independent predictors of metastasis risk estimation in high-risk for metastasis osteosarcoma patients [36]. Lastly, T2-weighted MRI radiomics provides reliable predictive markers for assessing chemotherapy response, survival, and disease-free outcomes in high-grade intramedullary osteosarcoma with an AUC of 0.708 ± 0.046 [37].

### 4.2. Tumor Detection

Tumor detection is the initial but critical step in improving patient outcomes through early diagnosis and timely intervention. Medical imaging modalities such as CT, MRI, and radiographs are generally preferred by radiologists due to their efficiency and ability to provide detailed information about the tumor’s structural insights (Table 2). Studies have shown that it is almost essential to follow pre-processing steps in order to enhance image quality before segmentation and make the tumor detection easier. For segmentation of cancerous regions, various techniques such as K-means clustering, Canny edge detection, and threshold-based methods have been applied with promising results, helping to reduce noise, define boundaries, and segment tumors effectively. Notably, AlexNet outperformed models like ResNet50 in early detection of parosteal osteosarcoma, osteochondroma, and enchondroma, using CT images and achieving a high testing accuracy of 100% [38].

CT–radiomics has a cobblestone role in differentiating benign and malignant tumors. By combining clinical features with radiomics, the model has a validation AUC of 0.823. This model is not only valuable for tumor detection but also for treatment planning [39].

Moreover, radiomics can be used in the pathology clinical workflow by assisting physicians in analyzing high-quality, low-noise images and reducing the intraclass variation between them. Deep learning is leading in tumor detection advancement through approaches that have been applied to histological analysis of osteosarcoma and addressing pathologists’ challenges, such as noise and intra-class variations [40].

Since the early detection of osteosarcoma, it remains critical for CNN to streamline the process. For instance, a comparative evaluation of four CNN-based models, VGG16, VGG19, DenseNet201, and ResNet101, revealed that ResNet101 was the most effective, achieving 90.36% accuracy, 89.51% precision, and an AUC of 0.9461. The model’s superior performance, coupled with efficient training time, highlights its power for osteosarcoma detection. The potential of advanced architectures like Xception, NASNetLarge, and EfficientNetV2L to further improve diagnostic accuracy and reliability, underscoring the transformative role of AI in early cancer detection [41]. The detection of osteosarcoma by a pathologist is a labor-intensive and difficult process that, on top of that, requires a lot of experience. Automatic detection systems like IF-FSM-C can detect osteosarcoma from whole slide images with an accuracy of 96.08% [42]. Similarly, a convolutional neural network (CNN) model demonstrated exceptional performance, achieving an accuracy of 99.8% in distinguishing normal from tumor images and 71.2% accuracy with a positive predictive value of 91.9% in differentiating benign from malignant bone tumors, offering a promising tool for histopathological diagnosis [43]. Additionally, the Bone Cancer Detection Network (BCDNet), a novel CNN-based model, achieved 96.29% accuracy for binary classification and 94.69% for multi-class classification, further underscoring its utility in early and accurate osteosarcoma detection [44].

Deep learning models further enhance diagnostic precision, with one designed for osteolytic osteosarcoma and giant cell tumor (GCT) on knee radiographs achieving 93.1% accuracy, significantly surpassing junior radiologists and performing comparably to senior radiologists [45]. Likewise, a deep learning model integrating biochemical markers such as alkaline phosphatase (ALP) and lactate dehydrogenase (LDH) with X-ray imaging features achieved 97.17% accuracy [46].

Moreover, a CNN model used in pediatric nuclear medicine holds promising results in differentiating benign and malignant bone disease on nuclear scintigraphy with an impressive accuracy of 96.17% and specificity of 91.67% [47]. On top of that, ChatGPT-4 (‘December 2023 version’) demonstrated high specificity of 100% for identifying bone lesions but showed limited sensitivity and accuracy for differentiating malignant from non-malignant conditions [48]. Furthermore, a deep learning model for detecting primary bone tumors on knee radiographs achieved 96.4% accuracy internally and 92.0% externally, significantly outperforming junior radiologists while being much faster [49].

Primary malignant bone tumors significantly affect not only adults but also the pediatric population, making early tumor detection important. A U-net-based AI model demonstrated remarkable sensitivity 95.52%, specificity 96.21%, on annotated X-ray data, outperforming traditional models and enhancing early detection and patient outcomes [50]. Similarly, DUconViT, a hybrid transformer–CNN system, achieved a Dice similarity coefficient of 92.4%, excelling in osteosarcoma segmentation and aiding surgical planning through efficient tumor size estimation [51]. Additionally, a Mask R-CNN model demonstrated 92% precision in distinguishing osteosarcoma and osteochondroma, further highlighting AI’s growing role in clinical diagnostics [52].

### 4.3. AI and Classification of PBT

The classification of primary bone tumors remains a significant challenge due to their rarity and diverse histological subtypes. The studies that focused on PBT classification are presented in Table 3. In their study, Song et al. employed a deep learning model to classify primary bone tumors using incomplete multimodal images from X-rays, CT, and MRI, and demonstrated a significant enhancement in classification accuracy. By integrating features from various imaging modalities, the model addressed the limitations of single-modality analysis and offered diagnostic support, scoring a satisfactory micro-average AUC of 0.847 [53]. Radiograph-based deep learning models have also been shown to improve radiologists’ performance in classifying histological types of primary bone tumors. A multicenter study of Xie et al. highlighted that integrating AI tools with radiologist expertise significantly enhanced diagnostic precision and efficiency with a macro average AUC of 0.904/0.873 [54]. Similarly, a preliminary study using deep learning-based classification of primary bone tumors on radiographs validated the potential of these models in clinical workflows in distinguishing between benign and non-benign AUC 0.894 and 0.877 and malignant and non-malignant 0.907 and 0.916 [55].

Advanced algorithms, such as the Remora optimization algorithm, have been utilized to enhance deep learning models for automated detection and classification of osteosarcoma; these methods demonstrated high accuracy and efficiency, making them valuable in early diagnosis and management [56]. Optimization techniques, including DenseNet and Elephant Herd optimization, have also been applied to classify osteosarcomas and giant cell tumors of the bone, with great success in handling complex imaging data [57,58]. Additionally, comprehensive diagnostic models for osteosarcoma classification using CT imaging features have been developed to address the specific challenges posed by these tumors, like the ones developed by Rahouma et al., a XG-Boost, support vector machine (SVM), and K-nearest neighbors, and Wang et al. showed that the principal component analysis (PCA- IPSO) outperforms traditional feature selection methods in predicting the accuracy of binary classification using support vector machine (SVM) [59,60].

Studies focusing on CT radiomics-based machine learning have effectively differentiated atypical cartilaginous tumors from chondrosarcomas, highlighting the power of texture analysis in tumor grading [61]. MRI radiomics-based models have further advanced classification efforts, particularly in distinguishing between low-grade and high-grade chondrosarcomas and other subtypes, through detailed texture and intensity mapping [62,63,64,65].

Several innovative optimization algorithms have been integrated into AI models for osteosarcoma classification. For instance, the Honey Badger optimization algorithm, combined with deep transfer learning, has been designed to achieve high diagnostic accuracy [66]. Similarly, a Bald Eagle Search Optimization integrated with an artificial neural network demonstrated promising results in osteosarcoma classification [67]. These methods underscore the importance of optimization in enhancing AI model performance.

Machine learning approaches have also been applied to classify and predict osteosarcoma grading. By leveraging metabolomic data alongside imaging, these models provided comprehensive diagnostic insights, further solidifying the utility of multimodal data integration [68]. A novel deep learning model called You Only Look Once (YOLO) for primary bone tumor detection and classification in full-field radiographs have also proven effective in handling large datasets, demonstrating their scalability and real-world applicability, detecting bone neoplasms from full-field radiographs in one shot and then simultaneously classify radiographs into normal, benign, intermediate, or malignant [69].

Multitask deep learning models have showcased their potential to simultaneously segment and classify primary bone tumors in radiographs, streamlining workflows and expediting diagnosis [70]. The application of AlexNet and ResNet architectures for spinal bone tumor classification has highlighted the versatility of AI in diverse clinical scenarios [71]. Systematic evaluations and meta-analyses have reinforced the diagnostic value of machine learning for malignant bone tumors, providing insights into its capabilities and limitations while guiding future research directions [72]. Advanced algorithms have also been developed for the segmentation and differentiation of pelvic and sacral osteosarcomas from Ewing’s sarcoma using CT-based machine learning networks [73,74].

X-rays radiomics-based models have shown promise in classifying atypical cartilaginous tumors and high-grade chondrosarcomas of long bones, further expanding the role of radiomics in bone tumor analysis [75,76]. Lastly, von Schacky et al. analyzed the radiographs from 934 patients over 20 years and successfully created a multitask DL model with an accuracy of 80.2%, which was higher than two radiology residents and comparable to two fellowship-trained radiologists, showing the high potential [77].

### 4.4. Tumor Segmentation

AI has shown significant potential to improve segmentation accuracy and efficiency in the management of primary bone tumors. All the selected studies that focused on tumor segmentation are shown in Table 4. Segmentation is necessary not only for preoperative plans but also can improve the tumor detection in AI applications. A systematic review of radiomics studies on chondrosarcoma reported strong diagnostic performance, with pooled DORs of 43.90 and AUCs between 0.90 and 0.94, but segmentation remains largely manual, reminding us of the need for AI integration in radiology workflow to increase our efficiency [78].

To address segmentation challenges in osteosarcoma, the ETUNet model achieves a Dice similarity coefficient (DSC) consistently above 90% and improving metrics like Intersection Over Union (IoU) and DSC pre-screening with the Slide Block Filter (SBF) demonstrated a robust accuracy of 95.67%, while noise reduction with the Non-Local Means (NLM) algorithm and CRF optimization further enhanced segmentation precision, proving highly strategic in image processing [79].

Likewise, SEAGNET uses supervised, edge-attention guidance to address blurred tumor boundaries, achieving outstanding metrics such as a DSC of 0.967, precision of 0.968, and accuracy of 0.996. Its ability to precisely localize malignant tumors significantly enhances diagnostic accuracy and clinical efficiency, making it a valuable tool, especially for high-grade primary bone tumors [80].

Additionally, the NSRDN framework, which integrates noise reduction through Differential Activation Filters (DAFs) and super resolution reconstruction, achieved 96.4% DSC, 92.8% IoU, and 95.5% accuracy using HRNet [81].

Recently, TBNet, a transformer-enhanced U-net model incorporating edge-enhanced modules and multi-head cross-fusion transformers, achieved a DSC of 0.949 and an accuracy of 0.997 in osteosarcoma MRI segmentation. Pre-screening with a Threshold Screening Filter (TSF) and noise reduction via fast NLM and Fourier transforms further supported this approach, optimizing segmentation accuracy while maintaining computational efficiency for early detection that can improve patients’ outcomes substantially [82].

Furthermore, the Eformer model combined with the DFANet segmentation network effectively addresses challenges like noise and blurred edges in osteosarcoma MRI images, achieving an accuracy of 0.995. This auxiliary segmentation method enhances tumor localization, precision, and automation, making it a cutting-edge tool for radiologists [83].

OSTransnet, which integrates U-net and transformer-based approaches with innovations like Channel-based transformers (CTrans) and Boundary Augmentation Blocks (BAB), achieved high tech metrics such as DSC of 0.949, IoU of 0.904, precision of 0.924, and recall of 0.981. These advancements enable faster, more accurate diagnoses while reducing physician workload, positioning OSTransnet as a promising tool for clinical applications [84].

BA-GCA Net incorporates modules like Grid Contextual Attention (GCA), Statistical Texture Learning Block (STLB), and Spatial Transformer Block (STB), achieving DSC of 0.927, IoU of 0.880, while maintaining low computational costs. These features make it effective for handling low-contrast, complex boundaries, improving diagnostic accuracy [85].

The 3D U-net model, trained using the MONAI framework, achieved mean DSC scores of 83.75% (T1-weighted), 85.45% (T2-weighted), and 87.62% (T1-gd) after preprocessing MRI images with techniques like Contrast-Limited Adaptive Histogram Equalization (CLAHE) and denoising filters, this approach demonstrated notable segmentation performance, effectively addressing blurred tumor edges and overfitting [86].

The DECIDE model leverages Multi-modality Feature Fusion and Recalibration (MFR), Lesion Attention Enhancement (LAE), and Boundary Context Aggregation (BCA) modules to improve segmentation performance, achieving precision of 74.85%, recall of 71.52%, DSC of 70.40%, and IoU of 54.50% [87].

The OSDCN framework, combining data preprocessing, segmentation with SepUNet, and conditional random fields (CRF), demonstrated DSC of 0.914, F1-score of 0.937, and IoU of 0.883. It relates to Mean Teacher optimization for noise reduction and multi-scale segmentation, enabling accurate tumor boundary delineation and area calculations on a dataset of over 80,000 MRI images, which demonstrates a reliable internal validation [88].

Manual and semiautomatic segmentation techniques using the GrowCut tool within the 3D-Slicer software version 4.6.2 achieved DSC ranging from 0.83 to 0.97 for manual segmentation and 0.71 to 0.96 for semiautomatic methods, with semiautomatic segmentation requiring significantly less time. These methods explain that semiautomatic approaches are more efficient but elaborate on the need for AI for reliability and reproducibility [89].

The MSRN (Multiple Supervised Residual Network) model further advanced CT-based segmentation with 89.22% DSC, 88.74% sensitivity, and 0.9305 F1-measure, demonstrating robust precision in mixed bone and soft tissue regions, making it an excellent tool to implement [90].

The OSGABN (Osteosarcoma Segmentation Guided Aggregated Bilateral Network) employs FaBiNet to integrate low-level and high-level contextual features, achieving 95% accuracy, DSC of 0.915, and IoU of 0.853 on a dataset of over 80,000 MRI images, making it highly applicable to resource-limited healthcare settings [91]. The U-net model for pediatric sarcoma segmentation in PET/CT scans achieved voxel-wise precision/sensitivity of 0.71/0.54 (thorax), 0.71/0.39 (extremities), and 0.52/0.38 (abdomen), despite challenges with high FDG uptake and limited training data, which promise that it can handle tumor variability and complex metabolic activity [92].

Additionally, a framework for bone cancer detection utilizing MRI images integrates preprocessing techniques like Alternate Sequential Filtering (ASF) and Decision-Based Median Filters (DBME-F), enhancing edge and texture retention while eliminating noise. The Modified DeeplabV3+ model with Atrous Spatial Pyramid Pooling (ASPP) enabled multi-scale feature analysis, achieving DSC of 70.40%, IoU of 54.50% [93].

The UATransNet framework, leveraging a modified U-net with self-attentive mechanisms and dense residual learning, achieved an IoU of 0.922 ± 0.03, DSC of 0.921 ± 0.04, and 96.2% accuracy, validated on 80,000 MRI images. The model efficiently mitigates noise and supports precise tumor edge detection, making it an optimal solution for osteosarcoma diagnosis [94].

RTUNet++, a hybrid architecture integrating ResNet, transformer attention mechanisms, and Dense Skip Connections, addressed challenges like spatial information loss and grayscale heterogeneity. Achieving a DSC of 0.82. Ablation studies confirmed the critical role of transformer blocks in segmentation performance, demonstrating RTUNet++’s potential for accurate segmentation in diverse tumor morphologies [95].

Among automated and semi-automated segmentation methods for osteosarcoma using diffusion-weighted MRI (DWI), SLIC-Superpixels (SLIC-S) and Fuzzy C-means clustering (FCM) achieved Dice coefficients (DC) of approximately 82% and 79%, respectively. These methods demonstrated rapid execution times and precision in delineating tumor regions, emphasizing their potential for advancing computer-aided diagnosis and treatment planning [96].

An integrated pipeline, incorporating the MPFNet model for segmentation, achieved a mean DSC of 84.19% and a high-quality segmentation rate (HQSR) of 94.38%, while its fusion nomogram predicted survival probabilities with a C-index of 0.806, surpassing traditional radiomics and clinical nomograms [97].

### 4.5. Insights into Discrimination and Future Steps by AI

Another area in which AI is demonstrating its potential is the discrimination between primary bone tumors by leveraging advanced imaging techniques and machine learning models to enhance differentiation accuracy. MRI-based texture analysis has demonstrated significant diagnostic value in distinguishing enchondroma from chondrosarcoma, as shown by Cilengir et al. that found Naive Bayes, K neighbors, and logistic regression models offered a non-invasive method for early and precise detection, achieving high accuracy and AUC for T1-weighted, FS-PD images and their combination, respectively [98]. Radiomics, combined with machine learning, has further refined the ability to distinguish between chondrosarcoma and enchondroma, as found by Erdem et al., emphasizing the potential of quantitative imaging features in tumor characterization with an advanced neural network that achieved a high diagnostic performance AUC of 0.979–0.984 [2]. Similarly, computed tomography (CT)-based machine learning networks have shown promise in automatically segmenting and differentiating pelvic and sacral osteosarcoma from Ewing’s sarcoma [99], enabling faster clinical decision-making. Deep learning algorithms, such as two-phase models, distinguish Ewing sarcoma from acute osteomyelitis in pediatric radiographs, achieving test accuracies of 90.6% and 86.7% in detecting pathological cases and differentiating Ewing sarcoma from osteomyelitis, respectively. Gradient-Weighted Class Activation Mapping (Grad-CAM) visualizations further validated these models by confirming their focus on clinically relevant regions [100]. Models like support vector machine and convolutional neural networks achieved impressive accuracies of 89.9% for SVM, 93.3% for CNNs, excellent and useful tools for assessing chemotherapy response and advancing personalized care [101].

Radiogenomics, functional imaging, and advanced surgical technologies are transforming the diagnosis and treatment of bone sarcomas, such as Ewing sarcoma. The integration of functional imaging with transcriptomics has revealed insights into tumor biology, such as glucose uptake patterns, aiding in personalized treatment approaches [102]. Innovations like single-shot multispectral quantitative phase imaging, enhanced by deep learning, and trained and validated on two different samples: the optical waveguide and MG63 osteosarcoma cells, allow for rapid, label-free visualization of biological samples, offering precise tumor characterization [103]. Lastly, computer-assisted tumor surgery (CATS) and 3D printing in surgical management provide improved preoperative planning and intraoperative accuracy, leading to better outcomes for bone sarcoma patients when implementing patient-specific instrumentation with custom-made implants [104].

### 4.6. Limitations of the Study

This narrative review is accompanied by some limitations that must be acknowledged. First, a direct comparison between the performance of the various AI models was not feasible due to the substantial heterogeneity in study designs, datasets, and reported metrics. While some studies reported AUC values, others utilized DSC, IoU, precision, or accuracy, making standardized comparisons challenging and limiting the ability to draw generalized conclusions on model superiority. A meta-analytic approach is urgently needed to establish standardized evaluation criteria and rank model performance across various imaging modalities and tumor types.

Second, overfitting remains a critical concern across many AI-based models included in this review, particularly in studies with small sample sizes or a lack of external validation. The majority of segmentation and classification algorithms were evaluated on institution-specific or limited datasets, raising concerns regarding generalizability, reproducibility, and robustness in diverse clinical settings. On top of that, some of them did not assess the external validity of the tools, which is crucial for real-world translation.

Finally, while this review focuses primarily on radiomics, ML, and DL, other valuable aspects such as model interpretability, medical error, data privacy, and ethical considerations were beyond the scope of our analysis but deserve attention in future investigations. In this context, the study by Shrivastava et al. (2023) emphasizes the importance of integrating feature extraction strategies with machine learning pipelines to enhance interpretability and clinical trust in AI-based decision systems for bone tumor diagnosis [105]. Their findings highlight the need for methodological rigor and transparency, reinforcing the need for reproducible AI research frameworks in musculoskeletal oncology.

## 5. Conclusions

AI has shown significant promise in advancing the diagnosis, classification, and treatment response prediction of primary malignant bone tumors (PBTs). From enhancing radiological interpretation to improving treatment outcomes through predictive modeling, AI represents a transformative tool in orthopedic oncology. The integration of advanced ML and DL techniques into clinical workflows has not only increased diagnostic accuracy but also enabled more personalized therapeutic approaches, potentially improving patient outcomes. However, achieving widespread clinical adoption will require further robust validation studies.

Despite its potential, our study highlights several limitations. Firstly, the application of AI to PBTs remains constrained by the rarity of these tumors, which limits the availability of high-quality, diverse datasets for model training and validation. Additionally, the lack of standardization in imaging protocols and radiomics feature extraction poses challenges to reproducibility and generalizability. Ethical considerations, including data privacy and the interpretability of complex AI models, also warrant careful attention. Future research should focus on addressing these limitations by fostering multicenter collaborations, developing explainable AI models, and integrating AI systems into clinical practice with robust regulatory oversight to ensure safety and efficacy.

## Figures and Tables

**Table 1:** Selected studies on AI-driven prediction and monitoring of treatment response in primary bone tumors.

Author	Year	Study Type	Imaging Modality	AI Model	Performance Metrics	Tumor Type
Gitto et al. [10]	2022	Retrospective	MRI	2D vs. 3D Radiomics	3D is superior in reproducibility	Ewing Sarcoma
Gitto et al. [11]	2022	Retrospective	MRI	3D Radiomics	Feature reproducibility in predicting NAC response	Ewing Sarcoma
Lin et al. [12]	2020	Retrospective	MRI	Delta-Radiomics	AUC 0.871 (train), 0.843 (validation)	Osteosarcoma
He et al. [13]	2022	Multicenter	MRI	LR, DT, SVM, NN	AUC 0.893	High-Grade Bone Sarcoma
Zhong et al. [14]	2022	Retrospective	MRI	DL + Radiomics Nomogram	AUC 0.793 (95% CI 0.610–0.975)	Osteosarcoma
Nie et al. [15]	2024	Retrospective	CT	DLRM	AUC 0.879 (95% CI 0.802–0.956)	Chondrosarcoma
Teo et al. [16]	2022	Retrospective	MRI	SVM (RBF)	Accuracy improved >95% with DCE-MRI	Osteosarcoma (Pediatric)
Ho et al. [17]	2020	Retrospective	MRI	Deep Interactive Learning (DIaL)	CNN training in 7 h	Osteosarcoma
Fu et al. [18]	2020	Retrospective	Histology (H&E)	Siamese Network (DS-Net)	Accuracy 95.1%	Osteosarcoma
Kim et al. [19]	2018	Retrospective	PET	DL + Radiomics	Higher prediction accuracy	Osteosarcoma
Hu et al. [20]	2021	Retrospective	DWI-MRI	CSDCNN	Better PSNR, MSE, EPI, accuracy, recall, F1, ADC stats	Osteosarcoma
Djuričić et al. [21]	2023	Retrospective	MRI	Fractal Radiomics + LASSO	AUC 0.95	Osteosarcoma
Zhang et al. [22]	2024	Retrospective	DWI-MRI	ML Radiomics Nomogram	AUC 0.848	Osteosarcoma
Huang et al. [23]	2020	Retrospective	Multi-parametric MRI	ML Model	AUCs: 0.93–0.97	Osteosarcoma
Zhang et al. [24]	2021	Retrospective	DCE-MRI	KNN, SVM, LR	AUCs: 0.86, 0.92, 0.93	Osteosarcoma
Zhang et al. [25]	2024	Retrospective	MRI	Radiomics (pre/post NAC)	AUC 0.999 (post), 0.915 (pre)	Osteosarcoma
Mori et al. [26]	2024	Retrospective	MRI (T1, T2)	Texture Analysis	AUCs 0.99 (T1), 0.94 (T2)	Osteosarcoma
Chen et al. [27]	2021	Multicenter	MRI	LASSO-LR	Radiomics signature prediction (no specific AUC reported)	Osteosarcoma
Miedler et al. [28]	2023	Retrospective	MRI	Radiomics	Predictive potential (no numerical metrics)	Ewing Sarcoma
Chaber et al. [29]	2019	Retrospective	IR Spectroscopy	ML	Accuracy 92%	Ewing Sarcoma
Dufau et al. [30]	2019	Retrospective	PET	ML + Radiomics	AUC 0.98, sensitivity 100%	Osteosarcoma
Jeong et al. [31]	2019	Retrospective	PET	Linear SVM + PCA	Improved AUC (no number)	Osteosarcoma
Bouhamama et al. [32]	2022	Retrospective	MRI	Radiomics	AUC 0.97	Osteosarcoma
Kim et al. [33]	2021	Retrospective	PET	CNN	Predictive (no numerical metrics)	Osteosarcoma
Helen et al. [34]	2024	Retrospective	PET	Binary CNN	Improved prediction	Osteosarcoma
Im et al. [35]	2017	Retrospective	PET	ML Using FDG-PET	Prognostic FDG-based features for NAC prediction	Osteosarcoma
Sheen et al. [36]	2019	Retrospective	PET	Logistic Model	SUVmax + GLZLM_SZLGE as predictors	Osteosarcoma
White et al. [37]	2023	Retrospective	T2 MRI	Radiomics	AUC 0.708 ± 0.046	High-Grade Osteosarcoma

**Table 2:** AI models are applied for the accurate detection of primary bone tumors in imaging datasets.

Author	Year	Study Type	Imaging Modality	AI Model	Performance Metrics	Tumor Type
Sampath et al. [38]	2024	Retrospective	CT	AlexNet	Accuracy 100%	Parosteal Osteosarcoma, Osteochondroma, Enchondroma
Sun et al. [39]	2021	Retrospective	CT	Radiomics + Clinical Model	AUC 0.823	Bone Tumors
Sanmartín et al. [40]	2024	Retrospective	Histology	FP-Growth + Transfer Learning + Stacking	Noise reduction and variation minimization	Osteosarcoma
Gawade et al. [41]	2023	Retrospective	MRI	ResNet101 (best among VGG16, VGG19, DenseNet)	Accuracy 90.36%, precision 89.51%, AUC 0.9461	Osteosarcoma
Bansal et al. [42]	2022	Retrospective	WSI	IF-FSM-C	Accuracy 96.08%	Osteosarcoma
Deng et al. [43]	2024	Retrospective	Histopathology	CNN	99.8% (normal vs. tumor), 71.2% (benign vs. malignant), PPV 91.9%	Bone Tumors
Rao et al. [44]	2024	Retrospective	Histology	BCDNet	Accuracy: 96.29% (binary), 94.69% (multi-class)	Bone Cancer
Shao et al. [45]	2024	Multicenter	X-Ray	DL model	Accuracy 93.1%	Osteosarcoma vs. GCT
Wang et al. [46]	2024	Retrospective	X-Ray + Labs	DL + ALP + LDH	Accuracy 97.17%	Osteosarcoma
Yang et al. [47]	2023	Retrospective	Nuclear Medicine	CNN	Accuracy 96.17%, specificity 91.67%	Pediatric Bone Disease
Ren et al. [48]	2024	Retrospective	X-Ray	ChatGPT-4	Specificity is 100%, but lower sensitivity	Osteosarcoma
Loraksa et al. [49]	2022	Retrospective	X-Ray	CNN	Accuracy 96.4% (internal), 92.0% (external)	Osteosarcoma
Hasei et al. [50]	2024	Retrospective	X-Ray	U-Net	Sensitivity 95.52%, specificity 96.21%	Pediatric Osteosarcoma
Ling et al. [51]	2022	Retrospective	MRI	DUconViT (Transformer + CNN)	Dice similarity coefficient 92.4%	Osteosarcoma
Xia et al. [52]	2023	Retrospective	X-Ray	Mask R-CNN	Precision 92%	Osteosarcoma, Osteochondroma

**Table 3:** AI-based classification systems for distinguishing primary bone tumor types in medical imaging.

Author	Year	Study Type	Imaging Modality	AI Model	Performance Metrics	Tumor Type
Song et al. [53]	2024	Retrospective	X-ray, CT, MRI	Multimodal DL Model	Micro-average AUC 0.847	Primary Bone Tumors
Xie et al. [54]	2024	Multicenter	Radiograph	DL + Radiologist	Macro-average AUC 0.904/0.873	Primary Bone Tumors
He et al. [55]	2020	Preliminary	Radiograph	DL Model	AUC: benign/non-benign 0.894/0.877; malignant 0.907/0.916	Primary Bone Tumors
Obaid et al. [56]	2023	Retrospective	CT	DL + Remora Optimization	High accuracy (not specified)	Osteosarcoma
He & Bi [57]	2024	Retrospective	MRI	Optimized DenseNet	Improved classification performance	Spinal Osteosarcoma vs. GCT
Malibari et al. [58]	2022	Retrospective	Image	Elephant Herd Optimization + DL	Effective classification	Osteosarcoma
Rahouma et al. [59]	2023	Retrospective	CT	XGBoost, SVM, KNN	Diagnostic model for osteosarcoma	Osteosarcoma
Wang et al. [60]	2024	Retrospective	CT	PCA-IPSO + SVM	Outperforms traditional feature selection	Osteosarcoma
Georgeanu et al. [61]	2021	Retrospective	MRI	CNN	Automated detection and classification	Bone Tumors
Sagar & Bhan [62]	2024	Retrospective	Not Specified	ML Model	Osteosarcoma grading classification	Osteosarcoma
Gitto et al. [63]	2019	Retrospective	MRI	Texture Analysis + ML	Low vs. high-grade chondrosarcoma classification	Chondrosarcoma
Gitto et al. [64]	2022	Retrospective	MRI	Radiomics + ML	ACT vs. grade II chondrosarcoma	Chondrosarcoma
Gitto et al. [65]	2020	Retrospective	MRI	Radiomics + ML	Bone chondrosarcoma classification	Chondrosarcoma
Vaiyapuri et al. [66]	2022	Retrospective	Image	Honey Badger Opt. + Transfer Learning	High diagnostic accuracy	Osteosarcoma
Jha et al. [67]	2022	Retrospective	MRI	Radiomic Signature	High vs. low-grade classification	Chondrosarcoma
Shen et al. [68]	2018	Retrospective	X-ray + Metabolomics	ML Model	Enhanced classification using combined features	Osteosarcoma
Li et al. [69]	2023	Retrospective	Full-field Radiograph	YOLO DL Model	Multi-class: normal, benign, intermediate, malignant	Primary Bone Tumors
Hadi et al. [70]	2023	Retrospective	Image	Bald Eagle Optimization + ANN	High accuracy	Osteosarcoma
Guo et al. [71]	2024	Retrospective	Radiograph	AlexNet and ResNet	Tumor malignancy classification	Spinal Bone Tumors
Li et al. [72]	2023	Meta-analysis	Multiple	ML Models	Diagnostic value confirmed	Malignant Bone Tumors
Gitto et al. [73]	2021	Retrospective	CT	Radiomics + ML	ACT vs. appendicular chondrosarcoma	Chondrosarcoma
Pan et al. [74]	2021	Retrospective	Radiograph	ML Model	Radiographic feature classification	Bone Tumors
Von Schacky et al. [75]	2022	Retrospective	X-Ray	ANN + RFC + GNB	AUC 0.79/0.90	Primary Bone Tumors
Gitto et al. [76]	2024	Retrospective	X-Ray	Radiomics + ML	ACT vs. high-grade chondrosarcoma	Chondrosarcoma
von Schacky et al. [77]	2021	Retrospective	Radiograph	Multitask DL	Accuracy 80.2%, better than residents, comparable to radiologists	Primary Bone Tumors

**Table 4:** Recent studies have utilized AI models for the segmentation of primary bone tumors in medical imaging.

Author	Year	Study Type	Imaging Modality	AI Model	Performance Metrics	Tumor Type
Zhong et al. [78]	2023	Systematic Review	MRI	Manual Segmentation	0.90–0.94 (AUC)	Chondrosarcoma
Wu et al. [79]	2022	Retrospective	MRI	ETUNet + SBF + NLM + CRF	DSC > 90%, Accuracy 95.67%	Osteosarcoma
Zhan et al. [80]	2023	Retrospective	MRI	SEAGNET	DSC 0.967, Accuracy 0.996	Bone Tumors
Zhong et al. [81]	2024	Retrospective	MRI	NSRDN with HRNet	DSC 96.4%, IoU 92.8%, Accuracy 95.5%	Osteosarcoma
Lv et al. [82]	2023	Retrospective	MRI	TBNet	DSC 0.949, Accuracy 0.997	Osteosarcoma
Wang et al. [83]	2022	Retrospective	MRI	Eformer + DFANet	Accuracy 0.995	Osteosarcoma
Liu et al. [84]	2022	Retrospective	MRI	OSTransNet	DSC 0.949, IoU 0.904	Osteosarcoma
Wu et al. [85]	2022	Retrospective	MRI	BA-GCA Net	DSC 0.927, IoU 0.880	Osteosarcoma
Lim et al. [86]	2023	Retrospective	MRI	3D U-Net (MONAI)	DSC 83.75–87.62%	Osteosarcoma
Wu et al. [87]	2024	Retrospective	MRI	DECIDE	DSC 70.40%, IoU 54.50%	Osteosarcoma
Wu et al. [88]	2022	Retrospective	MRI	OSDCN (SepUNet + CRF)	DSC 0.914, IoU 0.883	Osteosarcoma
Dionísio et al. [89]	2020	Retrospective	MRI	Manual and Semi-Automatic	DSC 0.71–0.97	Bone Sarcomas
Zhang et al. [90]	2018	Retrospective	CT	MSRN	DSC 89.22%, F1 0.9305	Osteosarcoma
Shen et al. [91]	2022	Retrospective	MRI	OSGABN (FaBiNet)	DSC 0.915, IoU 0.853	Osteosarcoma
Ørum et al. [92]	2019	Retrospective	PET/CT	U-Net	Precision 0.71, sensitivity 0.39–0.54	Pediatric Sarcoma
Kaur et al. [93]	2024	Retrospective	MRI	Modified DeepLabV3+ (ASPP)	DSC 70.40%, IoU 54.50%	Bone Cancer
Ouyang et al. [94]	2022	Retrospective	MRI	UATransNet	DSC 0.921, IoU 0.922	Osteosarcoma
Zou et al. [95]	2023	Retrospective	MRI	RTUNet++	DSC 0.82	Osteosarcoma
Kayal et al. [96]	2020	Retrospective	DWI-MRI	SLIC-S and FCM	DSC ~82%, ~79%	Osteosarcoma
Zhou et al. [97]	2024	Retrospective	MRI	MPFNet	DSC 84.19%, HQSR 94.38%	Osteosarcoma

## Abbreviations

The following abbreviations are used in this manuscript:

AI	Artificial Intelligence
PBT	Primary Bone Tumors
NAC	Neoadjuvant Chemotherapy
ML	Machine Learning
DL	Deep Learning
RS	Radiomics Signature
RBF	Radial Basis Function
CNN	Convolutional Neural Networks
MRI	Magnetic Resonance Imaging
AUC	Area Under the Curve
DT	Decision Tree
LR	Logic Recession
SVM	Support Vector Machine
DCA	Decision Curve Analysis
DLRM	Deep Learning Radiomics Model
DIaL	Deep Learning Interactive Model
DS-Net	Deep Supervision Network
H&E	Hematoxylin and Eosin
18F-FDG	Fluorine 18 Fluorodeoxyglucose
PET	Positron Emission Tomography
DWI	Diffusion-Weighted Imaging
PSNR	Peak Signal-to-Noise Ratio
MSE	Mean Squared Error
EPI	Edge Presence Index
LASSO	Least Absolute Shrinkage and Selection Operator
ROI	Region Of Interest
T2WI	T2 Weighted Imaging
T1CE	T1 Weighted Contrast-Enhanced Imaging
KNN	K Nearest Neighbor
NAC	Neoadjuvant Chemotherapy
DCE-MRI	Dynamic Contrast-Enhanced Magnetic Resonance Imaging
SUVmax	Maximum Standardized Uptake Value
CT	Computed Tomography
VGG16	Visual Geometry Group 16-layer Network
VGG19	Visual Geometry Group 19-layer Network
DenseNet201	Densely Connected Convolutional Network 201 Layers
ResNet101	Residual Network 101 Layers
NASNetLarge	Neural Architecture Search Network Large
EfficientNetV2L	Efficient Network Version 2 Large
IF-FSM-C	Inception Framework with Feature Selection Mechanism for Classification
BCDNet	Bone Cancer Detection Network
GCT	Giant Cell Tumor
ALP	Alkaline Phosphatase
LDH	Lactate Dehydrogenase
ChatGPT-4	Chat Generative Pre-trained Transformer 4
U-net	U-shaped Convolutional Network
DUconViT	Dual Convolutional Vision Transformer
Mask R-CNN	Mask Region-Based Convolutional Neural Network
PCA-IPSO	Principal Component Analysis Improved Particle Swarm Optimization
DECIDE	Deep Ensemble Classifier with Integration of Dual Enhancers
Grad-CAM	Gradient-weighted Class Activation Mapping
CATS	Computer-Assisted Tumor Surgery

## References

[1] Vogrin M., Trojner T., Kelc R. (2020). Artificial Intelligence in Musculoskeletal Oncological Radiology. Radiol. Oncol..

[2] Erdem F., Tamsel İ., Demirpolat G. (2023). The Use of Radiomics and Machine Learning for the Differentiation of Chondrosarcoma from Enchondroma. J. Clin. Ultrasound.

[3] Meng Y., Yang Y., Hu M., Zhang Z., Zhou X. (2023). Artificial Intelligence-Based Radiomics in Bone Tumors: Technical Advances and Clinical Application. Semin. Cancer Biol..

[4] Ye Q., Yang H., Lin B., Wang M., Song L., Xie Z., Lu Z., Feng Q., Zhao Y. (2024). Automatic Detection, Segmentation, and Classification of Primary Bone Tumors and Bone Infections Using an Ensemble Multi-Task Deep Learning Framework on Multi-Parametric MRIs: A Multi-Center Study. Eur. Radiol..

[5] Emil N.S., Sibbitt R.R., Sibbitt W.L. (2023). Machine Learning and Magnetic Resonance Imaging: Differentiating Benign from Malignant Osseous Tumors. J. Clin. Ultrasound.

[6] Yildirim M., Yildirim H. (2024). CT Radiomics-Based Machine Learning Model for Differentiating between Enchondroma and Low-Grade Chondrosarcoma. Med. Baltim..

[7] Zheng F., Yin P., Liang K., Wang Y., Hao W., Hao Q., Hong N. (2024). Fusion Radiomics-Based Prediction of Response to Neoadjuvant Chemotherapy for Osteosarcoma. Acad. Radiol..

[8] Avery E., Sanelli P.C., Aboian M., Payabvash S. (2022). Radiomics: A Primer on Processing Workflow and Analysis. Semin. Ultrasound CT MRI.

[9] Li M.D., Ahmed S.R., Choy E., Lozano-Calderon S.A., Kalpathy-Cramer J., Chang C.Y. (2022). Artificial Intelligence Applied to Musculoskeletal Oncology: A Systematic Review. Skelet. Radiol..

[10] Gitto S., Corino V., Bologna M., Marzorati L., Milazzo Machado E., Albano D., Messina C., Mainardi L., Sconfienza L.M. (2022). MRI Radiomics-Based Machine Learning to Predict Neoadjuvant Chemotherapy Response in Ewing Sarcoma. Insights Imaging.

[11] Gitto S., Corino V.D.A., Annovazzi A., Milazzo Machado E., Bologna M., Marzorati L., Albano D., Messina C., Serpi F., Anelli V. (2022). 3D vs. 2D MRI Radiomics in Skeletal Ewing Sarcoma: Feature Reproducibility and Preliminary Machine Learning Analysis on Neoadjuvant Chemotherapy Response Prediction. Front. Oncol..

[12] Lin P., Yang P.F., Chen S., Shao Y.Y., Xu L., Wu Y., Teng W., Zhou X.Z., Li B.H., Luo C. (2020). A Delta-Radiomics Model for Preoperative Evaluation of Neoadjuvant Chemotherapy Response in High-Grade Osteosarcoma. Cancer Imaging.

[13] He F., Xie L., Sun X., Xu J., Li Y., Liu R., Sun K., Shen D., Gu J., Ji T. (2022). A Scoring System for Predicting Neoadjuvant Chemotherapy Response in Primary High-Grade Bone Sarcomas: A Multicenter Study. Orthop. Surg..

[14] Zhong J., Zhang C., Hu Y., Zhang J., Liu Y., Si L., Xing Y., Ding D., Geng J., Jiao Q. (2022). Automated Prediction of the Neoadjuvant Chemotherapy Response in Osteosarcoma with Deep Learning and an MRI-Based Radiomics Nomogram. Eur. Radiol..

[15] Nie P., Zhao X., Ma J., Wang Y., Li B., Li X., Li Q., Xu Y., Dai Z., Wu J. (2024). Can the Preoperative CT-Based Deep Learning Radiomics Model Predict Histologic Grade and Prognosis of Chondrosarcoma?. Eur. J. Radiol..

[16] Teo K.Y., Daescu O., Cederberg K., Sengupta A., Leavey P.J. (2022). Correlation of Histopathology and Multi-Modal Magnetic Resonance Imaging in Childhood Osteosarcoma: Predicting Tumor Response to Chemotherapy. PLoS ONE.

[17] Ho D.J., Agaram N.P., Schüffler P.J., Vanderbilt C.M., Jean M.-H., Hameed M.R., Fuchs T.J. (2020). Deep Interactive Learning: An Efficient Labeling Approach for Deep Learning-Based Osteosarcoma Treatment Response Assessment. Medical Image Computing and Computer Assisted Intervention–MICCAI 2020, Proceedings of the 23rd International Conference, Lima, Peru, 4–8 October 2020.

[18] Fu Y., Xue P., Ji H., Cui W., Dong E. (2020). Deep Model with Siamese Network for Viable and Necrotic Tumor Regions Assessment in Osteosarcoma. Med. Phys..

[19] Kim W., Park J., Sheen H., Byun B.H., Lim I., Kong C.-B., Lim S.M., Woo S.-K. (2018). Development of Deep Learning Model for Prediction of Chemotherapy Response Using PET Images and Radiomics Features. Proceedings of the 2018 IEEE Nuclear Science Symposium and Medical Imaging Conference Proceedings (NSS/MIC).

[20] Hu Y., Tang J., Zhao S., Li Y. (2021). Diffusion-Weighted Imaging-Magnetic Resonance Imaging Information under Class-Structured Deep Convolutional Neural Network Algorithm in the Prognostic Chemotherapy of Osteosarcoma. Sci. Program..

[21] Djuričić G.J., Ahammer H., Rajković S., Kovač J.D., Milošević Z., Sopta J.P., Radulovic M. (2023). Directionally Sensitive Fractal Radiomics Compatible With Irregularly Shaped Magnetic Resonance Tumor Regions of Interest: Association With Osteosarcoma Chemoresistance. J. Magn. Reson. Imaging.

[22] Zhang L., Gao Q., Dou Y., Cheng T., Xia Y., Li H., Gao S. (2024). Evaluation of the Neoadjuvant Chemotherapy Response in Osteosarcoma Using the MRI DWI-Based Machine Learning Radiomics Nomogram. Front. Oncol..

[23] Huang B., Wang J., Sun M., Chen X., Xu D., Li Z.P., Ma J., Feng S.T., Gao Z. (2020). Feasibility of Multi-Parametric Magnetic Resonance Imaging Combined with Machine Learning in the Assessment of Necrosis of Osteosarcoma after Neoadjuvant Chemotherapy: A Preliminary Study. BMC Cancer.

[24] Zhang L., Ge Y., Gao Q., Zhao F., Cheng T., Li H., Xia Y. (2021). Machine Learning-Based Radiomics Nomogram With Dynamic Contrast-Enhanced MRI of the Osteosarcoma for Evaluation of Efficacy of Neoadjuvant Chemotherapy. Front. Oncol..

[25] Zhang Y., Zhi L., Li J., Wang M., Chen G., Yin S. (2024). Magnetic Resonance Imaging Radiomics Predicts Histological Response to Neoadjuvant Chemotherapy in Localized High-Grade Osteosarcoma of the Extremities. Acad. Radiol..

[26] Mori Y., Ren H., Mori N., Watanuki M., Hitachi S., Watanabe M., Mugikura S., Takase K. (2024). Magnetic Resonance Imaging Texture Analysis Based on Intraosseous and Extraosseous Lesions to Predict Prognosis in Patients with Osteosarcoma. Diagnostics.

[27] Chen H., Zhang X., Wang X., Quan X., Deng Y., Lu M., Wei Q., Ye Q., Zhou Q., Xiang Z. (2021). MRI-Based Radiomics Signature for Pretreatment Prediction of Pathological Response to Neoadjuvant Chemotherapy in Osteosarcoma: A Multicenter Study. Eur. Radiol..

[28] Miedler J., Schaal M., Götz M., Cario H., Beer M. (2023). Potential Role of MRI-Based Radiomics in Prediction of Chemotherapy Response in Pediatric Patients with Ewing-Sarcoma. Pediatr. Radiol..

[29] Chaber R., Arthur C.J., Łach K., Raciborska A., Michalak E., Bilska K., Drabko K., Depciuch J., Kaznowska E., Cebulski J. (2019). Predicting Ewing Sarcoma Treatment Outcome Using Infrared Spectroscopy and Machine Learning. Molecules.

[30] Dufau J., Bouhamama A., Leporq B., Malaureille L., Beuf O., Gouin F., Pilleul F., Marec-Berard P. (2019). Prediction of Chemotherapy Response in Primary Osteosarcoma Using the Machine Learning Technique on Radiomic Data. Bull. Cancer.

[31] Jeong S.Y., Kim W., Byun B.H., Kong C.B., Song W.S., Lim I., Lim S.M., Woo S.K. (2019). Prediction of Chemotherapy Response of Osteosarcoma Using Baseline (18)F-FDG Textural Features Machine Learning Approaches with PCA. Contrast Media Mol. Imaging.

[32] Bouhamama A., Leporq B., Khaled W., Nemeth A., Brahmi M., Dufau J., Marec-Bérard P., Drapé J.L., Gouin F., Bertrand-Vasseur A. (2022). Prediction of Histologic Neoadjuvant Chemotherapy Response in Osteosarcoma Using Pretherapeutic MRI Radiomics. Radiol. Imaging Cancer.

[33] Kim J., Jeong S.Y., Kim B.C., Byun B.H., Lim I., Kong C.B., Song W.S., Lim S.M., Woo S.K. (2021). Prediction of Neoadjuvant Chemotherapy Response in Osteosarcoma Using Convolutional Neural Network of Tumor Center (18)F-FDG PET Images. Diagnostics.

[34] Helen R., Gurumoorthy G., Thennarasu S.R., Sakthivel P.R. (2024). Prediction of Osteosarcoma Using Binary Convolutional Neural Network: A Machine Learning Approach. Proceedings of the 2024 Second International Conference on Emerging Trends in Information.

[35] Im H.-J., McIlwain S., Ong I., Lee I., Song C., Shulkin B., Cho S. (2017). Prediction of Response to Neoadjuvant Chemotherapy Using Machine Learning Algorithm Trained by Baseline FDG-PET Textural Parameters in Osteosarcoma. J. Nucl. Med..

[36] Sheen H., Kim W., Byun B.H., Kong C.-B., Lim I., Lim S.M., Woo S.-K. (2019). Prognostic and Predictive Logistic Model for Osteosarcoma Using Metabolic Imaging Phenotypes. J. Nucl. Med..

[37] White L.M., Atinga A., Naraghi A.M., Lajkosz K., Wunder J.S., Ferguson P., Tsoi K., Griffin A., Haider M. (2023). T2-Weighted MRI Radiomics in High-Grade Intramedullary Osteosarcoma: Predictive Accuracy in Assessing Histologic Response to Chemotherapy, Overall Survival, and Disease-Free Survival. Skelet. Radiol..

[38] Sampath K., Rajagopal S., Chintanpalli A. (2024). A Comparative Analysis of CNN-Based Deep Learning Architectures for Early Diagnosis of Bone Cancer Using CT Images. Sci. Rep..

[39] Sun W., Liu S., Guo J., Hao D., Hou F., Wang H., Xu W. (2021). A CT-Based Radiomics Nomogram for Distinguishing between Benign and Malignant Bone Tumours. Cancer Imaging.

[40] Sanmartín J., Azuero P., Hurtado R. (2024). A Modern Approach to Osteosarcoma Tumor Identification Through Integration of FP-Growth, Transfer Learning and Stacking Model. International Conference on Information Technology & Systems.

[41] Gawade S., Bhansali A., Patil K., Shaikh D. (2023). Application of the Convolutional Neural Networks and Supervised Deep-Learning Methods for Osteosarcoma Bone Cancer Detection. Healthc. Anal..

[42] Bansal P., Gehlot K., Singhal A., Gupta A. (2022). Automatic Detection of Osteosarcoma Based on Integrated Features and Feature Selection Using Binary Arithmetic Optimization Algorithm. Multimed. Tools Appl..

[43] Deng S., Huang Y., Li C., Qian J., Wang X. (2024). Auxiliary Diagnosis of Primary Bone Tumors Based on Machine Learning Model. J. Bone Oncol..

[44] Rao B.D., Madhavi K. (2024). BCDNet: A Deep Learning Model with Improved Convolutional Neural Network for Efficient Detection of Bone Cancer Using Histology Images. Int. J. Comput. Exp. Sci. Eng..

[45] Shao J., Lin H., Ding L., Li B., Xu D., Sun Y., Guan T., Dai H., Liu R., Deng D. (2024). Deep Learning for Differentiation of Osteolytic Osteosarcoma and Giant Cell Tumor around the Knee Joint on Radiographs: A Multicenter Study. Insights Imaging.

[46] Wang S., Shen Y., Zeng F., Wang M., Li B., Shen D., Tang X., Wang B. (2024). Exploiting Biochemical Data to Improve Osteosarcoma Diagnosis with Deep Learning. Health Inf. Sci. Syst..

[47] Yang P., Jiang L., Xiang Y., Wei J., Zhao Z., Cai H., Yi Z., Li L. (2023). Deep-Learning Model for Differentiation of Pediatric Bone Diseases by Bone Scintigraphy: A Feasibility Study. Eur. J. Nucl. Med. Mol. Imaging.

[48] Ren Y., Guo Y., He Q., Cheng Z., Huang Q., Yang L. (2024). Exploring Whether ChatGPT-4 with Image Analysis Capabilities Can Diagnose Osteosarcoma from X-Ray Images. Exp. Hematol. Oncol..

[49] Loraksa C., Mongkolsomlit S., Nimsuk N., Uscharapong M., Kiatisevi P. (2022). Effectiveness of Learning Systems from Common Image File Types to Detect Osteosarcoma Based on Convolutional Neural Networks (CNNs) Models. J. Imaging.

[50] Hasei J., Nakahara R., Otsuka Y., Nakamura Y., Hironari T., Kahara N., Miwa S., Ohshika S., Nishimura S., Ikuta K. (2024). High-Quality Expert Annotations Enhance Artificial Intelligence Model Accuracy for Osteosarcoma X-Ray Diagnosis. Cancer Sci..

[51] Ling Z., Yang S., Gou F., Dai Z., Wu J. (2022). Intelligent Assistant Diagnosis System of Osteosarcoma MRI Image Based on Transformer and Convolution in Developing Countries. IEEE J. Biomed. Health Inf..

[52] Xia G., Ran T., Wu H., Wang M., Pan J. (2023). The Development of Mask R-CNN to Detect Osteosarcoma and Oste-Ochondroma in X-Ray Radiographs. Comput. Methods Biomech. Biomed. Eng. Imaging Vis..

[53] Song L., Li C., Tan L., Wang M., Chen X., Ye Q., Li S., Zhang R., Zeng Q., Xie Z. (2024). A Deep Learning Model to Enhance the Classification of Primary Bone Tumors Based on Incomplete Multimodal Images in X-Ray, CT, and MRI. Cancer Imaging.

[54] Xie Z., Zhao H., Song L., Ye Q., Zhong L., Li S., Zhang R., Wang M., Chen X., Lu Z. (2024). A Radiograph-Based Deep Learning Model Improves Radiologists’ Performance for Classification of Histological Types of Primary Bone Tumors: A Multicenter Study. Eur. J. Radiol..

[55] He Y., Pan I., Bao B., Halsey K., Chang M., Liu H., Peng S., Sebro R.A., Guan J., Yi T. (2020). Deep Learning-Based Classification of Primary Bone Tumors on Radiographs: A Preliminary Study. EBioMedicine.

[56] Obaid M.K., Abed H.A., Abdullah S.B., Al-Jawahry H.M., Majed S., Hassan A.R. (2023). Automated Osteosarcoma Detection and Classification Using Advanced Deep Learning with Remora Optimization Algorithm. Proceedings of the 2023 6th International Conference on Engineering Technology and its Applications (IICETA).

[57] He J., Bi X. (2024). Automatic Classification of Spinal Osteosarcoma and Giant Cell Tumor of Bone Using Optimized DenseNet. J. Bone Oncol..

[58] Malibari A.A., Alzahrani J.S., Obayya M., Negm N., Al-Hagery M.A., Salama A.S., Hilal A.M. (2022). Biomedical Osteosarcoma Image Classification Using Elephant Herd Optimization and Deep Learning. Comput. Mater. Contin..

[59] Rahouma K.H., Abdellatif A.S. (2023). Bone Osteosarcoma Tumor Classification. Indones. J. Electr. Eng. Comput. Sci..

[60] Wang Y., Wang Z., Zhang B., Yang F. (2024). Comprehensive Diagnostic Model for Osteosarcoma Classification Using CT Imaging Features. J. Bone Oncol..

[61] Georgeanu V., Mamuleanu M.-L., Selisteanu D. (2021). Convolutional Neural Networks for Automated Detection and Classification of Bone Tumors in Magnetic Resonance Imaging. Proceedings of the 2021 IEEE International Conference on Artificial Intelligence, Robotics, and Communication (ICAIRC).

[62] Sagar C.V., Bhan A. (2024). Machine Learning Approach to Classify and Predict Osteosarcoma Grading. Proceedings of the 2024 International Conference on Automation and Computation (AUTOCOM).

[63] Gitto S., Albano D., Chianca V., Cuocolo R., Ugga L., Messina C., Sconfienza L.M. (2019). Machine Learning Classification of Low-Grade and High-Grade Chondrosarcomas Based on MRI-Based Texture Analysis. Semin. Musculoskelet. Radiol..

[64] Gitto S., Cuocolo R., van Langevelde K., van de Sande M.A.J., Parafioriti A., Luzzati A., Imbriaco M., Sconfienza L.M., Bloem J.L. (2022). MRI Radiomics-Based Machine Learning Classification of Atypical Cartilaginous Tumour and Grade II Chondrosarcoma of Long Bones. EBioMedicine.

[65] Gitto S., Cuocolo R., Albano D., Chianca V., Messina C., Gambino A., Ugga L., Cortese M.C., Lazzara A., Ricci D. (2020). MRI Radiomics-Based Machine-Learning Classification of Bone Chondrosarcoma. Eur. J. Radiol..

[66] Vaiyapuri T., Jothi A., Narayanasamy K., Kamatchi K., Kadry S., Kim J. (2022). Design of a Honey Badger Optimization Algorithm with a Deep Transfer Learning-Based Osteosarcoma Classification Model. Cancers.

[67] Jha A.K., Nayak P., Mithun S., Sherkhane U., Jaiswar V., Nath B., Tripathi A., Mehta G.M., Panchal S., Purandare N. (2022). Development and Validation of Radiomic Signature for Classification of High and Low-Grade Chondrosarcoma: A Pilot Study. Mol. Imaging Biol..

[68] Shen R., Li Z., Zhang L., Hua Y., Mao M., Cai Z., Qiu Y., Gryak J., Najarian K. (2018). Osteosarcoma Patients Classification Using Plain X-Rays and Metabolomic Data. Annu. Int. Conf. IEEE Eng. Med. Biol. Soc..

[69] Li J., Li S., Li X., Miao S., Dong C., Gao C., Liu X., Hao D., Xu W., Huang M. (2023). Primary Bone Tumor Detection and Classification in Full-Field Bone Radiographs via YOLO Deep Learning Model. Eur. Radiol..

[70] Hadi M.R., Hassan A.R., Mohammed I.H., Alazzai W.K., Alzubaidi L.H., Ai Sadi H.I. (2023). Integrated Design of Artificial Neural Network with Bald Eagle Search Optimization for Osteosarcoma Classification. Proceedings of the 2023 6th International Conference on Engineering Technology and its Applications (IICETA).

[71] Guo C., Chen Y., Li J. (2024). Radiographic Imaging and Diagnosis of Spinal Bone Tumors: AlexNet and ResNet for the Classification of Tumor Malignancy. J. Bone Oncol..

[72] Li Y., Dong B., Yuan P. (2023). The Diagnostic Value of Machine Learning for the Classification of Malignant Bone Tumor: A Systematic Evaluation and Meta-Analysis. Front. Oncol..

[73] Gitto S., Cuocolo R., Annovazzi A., Anelli V., Acquasanta M., Cincotta A., Albano D., Chianca V., Ferraresi V., Messina C. (2021). CT Radiomics-Based Machine Learning Classification of Atypical Cartilaginous Tumours and Appendicular Chondrosarcomas. EBioMedicine.

[74] Pan D., Liu R., Zheng B., Yuan J., Zeng H., He Z., Luo Z., Qin G., Chen W. (2021). Using Machine Learning to Unravel the Value of Radiographic Features for the Classification of Bone Tumors. BioMed Res. Int..

[75] von Schacky C.E., Wilhelm N.J., Schäfer V.S., Leonhardt Y., Jung M., Jungmann P.M., Russe M.F., Foreman S.C., Gassert F.G., Gassert F.T. (2022). Development and Evaluation of Machine Learning Models Based on X-Ray Radiomics for the Classification and Differentiation of Malignant and Benign Bone Tumors. Eur. Radiol..

[76] Gitto S., Annovazzi A., Nulle K., Interlenghi M., Salvatore C., Anelli V., Baldi J., Messina C., Albano D., Di Luca F. (2024). X-Rays Radiomics-Based Machine Learning Classification of Atypical Cartilaginous Tumour and High-Grade Chondrosarcoma of Long Bones. EBioMedicine.

[77] von Schacky C.E., Wilhelm N.J., Schäfer V.S., Leonhardt Y., Gassert F.G., Foreman S.C., Gassert F.T., Jung M., Jungmann P.M., Russe M.F. (2021). Multitask Deep Learning for Segmentation and Classification of Primary Bone Tumors on Radiographs. Radiology.

[78] Zhong J., Hu Y., Ge X., Xing Y., Ding D., Zhang G., Zhang H., Yang Q., Yao W. (2023). A Systematic Review of Radiomics in Chondrosarcoma: Assessment of Study Quality and Clinical Value Needs Handy Tools. Eur. Radiol..

[79] Wu J., Xiao P., Huang H., Gou F., Zhou Z., Dai Z. (2022). An Artificial Intelligence Multiprocessing Scheme for the Diagnosis of Osteosarcoma MRI Images. IEEE J. Biomed. Health Inf..

[80] Zhan X., Liu J., Long H., Zhu J., Tang H., Gou F., Wu J. (2023). An Intelligent Auxiliary Framework for Bone Malignant Tumor Lesion Segmentation in Medical Image Analysis. Diagnostics.

[81] Zhong X., Gou F., Wu J. (2024). An Intelligent MRI Assisted Diagnosis and Treatment System for Osteosarcoma Based on Super-Resolution. Complex Intell. Syst..

[82] Lv B., Liu F., Li Y., Nie J., Gou F., Wu J. (2023). Artificial Intelligence-Aided Diagnosis Solution by Enhancing the Edge Features of Medical Images. Diagnostics.

[83] Wang L., Yu L., Zhu J., Tang H., Gou F., Wu J. (2022). Auxiliary Segmentation Method of Osteosarcoma in MRI Images Based on Denoising and Local Enhancement. Healthcare.

[84] Liu F., Zhu J., Lv B., Yang L., Sun W., Dai Z., Gou F., Wu J. (2022). Auxiliary Segmentation Method of Osteosarcoma MRI Image Based on Transformer and U-Net. Comput. Intell. Neurosci..

[85] Wu J., Liu Z., Gou F., Zhu J., Tang H., Zhou X., Xiong W. (2022). BA-GCA Net: Boundary-Aware Grid Contextual Attention Net in Osteosarcoma MRI Image Segmentation. Comput. Intell. Neurosci..

[86] Lim C.C., Ling A.H.W., Chong Y.F., Mashor M.Y., Alshantti K., Aziz M.E. (2023). Comparative Analysis of Image Processing Techniques for Enhanced MRI Image Quality: 3D Reconstruction and Segmentation Using 3D U-Net Architecture. Diagnostics.

[87] Wu Y., Li J., Wang X., Zhang Z., Zhao S. (2024). DECIDE: A Decoupled Semantic and Boundary Learning Network for Precise Osteosarcoma Segmentation by Integrating Multi-Modality MRI. Comput. Biol. Med..

[88] Wu J., Yang S., Gou F., Zhou Z., Xie P., Xu N., Dai Z. (2022). Intelligent Segmentation Medical Assistance System for MRI Images of Osteosarcoma in Developing Countries. Comput. Math. Methods Med..

[89] Dionísio F.C.F., Oliveira L.S., Hernandes M.A., Engel E.E., Rangayyan R.M., Azevedo-Marques P.M., Nogueira-Barbosa M.H. (2020). Manual and Semiautomatic Segmentation of Bone Sarcomas on MRI Have High Similarity. Braz. J. Med. Biol. Res..

[90] Zhang R., Huang L., Xia W., Zhang B., Qiu B., Gao X. (2018). Multiple Supervised Residual Network for Osteosarcoma Segmentation in CT Images. Comput. Med. Imaging Graph..

[91] Shen Y., Gou F., Dai Z. (2022). Osteosarcoma MRI Image-Assisted Segmentation System Base on Guided Aggregated Bilateral Network. Mathematics.

[92] Ørum L., Banke K., Borgwardt L., Hansen A., Højgaard L., Andersen F., Ladefoged C. (2019). Pediatric Sarcoma Segmentation Using Deep Learning. J. Nucl. Med..

[93] Kaur C., Grag U. (2024). Preprocessing and Segmentation of MRI Images for Bone Cancer Detection Using Aurous Spatial Pooling With Deeplabv3. Grenze Sci. Soc..

[94] Ouyang T., Yang S., Gou F., Dai Z., Wu J. (2022). Rethinking U-Net from an Attention Perspective with Transformers for Osteosarcoma MRI Image Segmentation. Comput. Intell. Neurosci..

[95] Zou B., Chen Y., Chen Z., Sun Y., Huang Y., Qin F., Wang C. (2023). RTUNet++: Assessment of Osteosarcoma MRI Image Segmentation Leveraging Hybrid CNN-Transformer Approach with Dense Skip Connection. Proceedings of the 2023 8th International Conference on Signal and Image Processing (ICSIP).

[96] Baidya Kayal E., Kandasamy D., Sharma R., Bakhshi S., Mehndiratta A. (2020). Segmentation of Osteosarcoma Tumor Using Diffusion Weighted MRI: A Comparative Study Using Nine Segmentation Algorithms. Signal Image Video Process.

[97] Zhou Z., Xie P., Dai Z., Wu J. (2024). Self-Supervised Tumor Segmentation and Prognosis Prediction in Osteosarcoma Using Multiparametric MRI and Clinical Characteristics. Comput. Methods Programs Biomed..

[98] Cilengir A.H., Evrimler S., Serel T.A., Uluc E., Tosun O. (2023). The Diagnostic Value of Magnetic Resonance Imaging-Based Texture Analysis in Differentiating Enchondroma and Chondrosarcoma. Skelet. Radiol..

[99] Yin P., Wang W., Wang S., Liu T., Sun C., Liu X., Chen L., Hong N. (2023). The Potential for Different Computed Tomography-Based Machine Learning Networks to Automatically Segment and Differentiate Pelvic and Sacral Osteosarcoma from Ewing’s Sarcoma. Quant. Imaging Med. Surg..

[100] Consalvo S., Hinterwimmer F., Neumann J., Steinborn M., Salzmann M., Seidl F., Lenze U., Knebel C., Rueckert D., Burgkart R.H.H. (2022). Two-Phase Deep Learning Algorithm for Detection and Differentiation of Ewing Sarcoma and Acute Osteomyelitis in Paediatric Radiographs. Anticancer Res..

[101] Arunachalam H.B., Mishra R., Daescu O., Cederberg K., Rakheja D., Sengupta A., Leonard D., Hallac R., Leavey P. (2019). Viable and Necrotic Tumor Assessment from Whole Slide Images of Osteosarcoma Using Machine-Learning and Deep-Learning Models. PLoS ONE.

[102] Prexler C., Kesper M.S., Mustafa M., Seemann W., Schmidt O., Gall K., Specht K., Rechl H., Knebel C., Woertler K. (2019). Radiogenomics in Ewing Sarcoma: Integration of Functional Imaging and Transcriptomics Characterizes Tumor Glucose Uptake. Eur. J. Nucl. Med. Mol. Imaging.

[103] Bhatt S., Butola A., Kumar A., Thapa P., Joshi A., Jadhav S., Singh N., Prasad D.K., Agarwal K., Mehta D.S. (2023). Single-Shot Multispectral Quantitative Phase Imaging of Biological Samples Using Deep Learning. Appl. Opt..

[104] McCulloch R.A., Frisoni T., Kurunskal V., Donati D.M., Jeys L. (2021). Computer Navigation and 3d Printing in the Surgical Management of Bone Sarcoma. Cells.

[105] Shrivastava A., Nag M.K. (2024). Enhancing Bone Cancer Diagnosis Through Image Extraction and Machine Learning: A State-of-the-Art Approach. Surg. Innov..

